# The Effect of Traditional and Stabilization-Oriented Exercises on Deep Stabilization System Function in Elite Futsal Players

**DOI:** 10.3390/sports8120153

**Published:** 2020-11-28

**Authors:** Radim Jebavy, Jiří Baláš, Helena Vomackova, Jakub Szarzec, Petr Stastny

**Affiliations:** 1 Department of Track and Field, Faculty of Physical Education and Sport, Charles University, 16252 Prague, Czech Republic; radim.jebavy@email.cz; 2 Sport Research Center, Faculty of Physical Education and Sport, Charles University, 16252 Prague, Czech Republic; balas@ftvs.cuni.cz; 3 Department of Physiotherapy, Faculty of Physical Education and Sport, Charles University, 16252 Prague, Czech Republic; hvomackova@ftvs.cuni.cz; 4 Faculty of Nursing and Professional Health Studies, Health University in Bratislava, 83101 Bratislava, Slovakia; szarzecjakub@gmail.com; 5 Department of Sport Games, Faculty of Physical Education and Sport, Charles University, 16252 Prague, Czech Republic

**Keywords:** strength training, complex exercises, trunk stability, injury prevention

## Abstract

Background: This study aimed to compare the effect of traditional and stability-oriented strength exercises on trunk stability and deep stabilization system (DSS) activation in elite futsal players. Methods: Twenty elite futsal players (21–34 years, 180 ± 13 cm, 79 ± 15 kg) were randomly divided into a group that performed stability-oriented exercises and a group that performed traditional strength exercises. Both interventions lasted for 10 weeks and included 25 strength training sessions. Main outcome measures: The DSS pretest and posttest included the diaphragm test, trunk flexion test, back extension test, hip flexion test, intraabdominal pressure test, and a side plank test on a 1–5 point scale. Results: Both groups had similar initial test results, where the stability-oriented exercise group had significantly improved intraabdominal pressure test (*p* = 0.004, by lower quartile rate), trunk flexion (*p* = 0.036, by 0.5 grade in median), and side plank (*p* = 0.002, by 1 grade in median) in posttest results. Traditional exercise did not change the results of any of the included DSS function tests. Conclusions: Stabilization-oriented exercises effectively activate the functions of the DSS and should be prioritized over traditional strength exercises in injury prevention training programs. The use of stabilization-oriented exercises might prevent injury and overloading in elite futsal players.

## 1. Introduction

Elite futsal is a physically high demanding sport with a high injury rate up to incidence frequency of 196 injuries per 1000 player match hours, where 70% are the injuries of the lower extremity [1,2]. This 2 × 20-min game is typical by high-intensity and intermittent actions of the player, including maximal sprint and agility effort [3,4,5] and covering a total distance of about 3749 m [6]. Thus, as the fatigue during maximal sprinting increases, there is also an increased risk of injury [7]. Therefore, injury prevention currently plays a key role in strength training and conditioning, where strengthening should be focused on trunk stability and activation of the deep stabilization system (DSS) without overloading superficial muscles [8,9,10].

The DSS includes muscle groups that ensure the stabilization of the spine during static and dynamic movements [11,12]. Previously, the deep spine extensors and flexors, musculi multifidi, diaphragm, pelvic floor muscles, and abdominal muscle were considered the main components of the DSS system [12]. The co-contraction of those muscles should increase intraabdominal pressure [13], which helps to stabilize the trunk [14,15] and might prevent vertebral disorders, such as vertebral disk herniation [16], soft tissue microtrauma, or injury. If the DSS is not activated, stabilization must be achieved by superficial muscle groups that have a limited ability to keep the spine in a neutral position, which usually results in muscle spasms, e.g., shortening of the lower back superficial spine extensors, and the occurrence of low back pain [12]. However, the evaluation of whether DSS is functioning is not obvious like in superficial muscles; therefore, extremely specific testing combining abdominal and back muscle functions with manual and palpation skills has been developed [11,12,17,18].

Although some complex exercises with light external loads are recommended for DSS muscle development [19,20], others, such as squats and sit-ups, have been reported as preferential for the superficial erector spinae [20] or rectus abdominis [8,21]. In exercise selection for athletes, it is crucial to focus on those exercises that develop the DSS muscles without overloading the superficial agonists [19], e.g., various quadruped and tripod stability positions [11,22]. This approach is in practice applied by the core training, training on unstable surfaces, or dynamic stabilization system training [23,24], where those approaches are typical by the positions challenging for stability, thus using different sources of stabilization-oriented exercises. On the other hand, traditional exercises that are performed on stable surfaces and without a high requirement for stability challenging positions have been reported for the improvements of functional movements [23,24], although with lower progress in improvement than stability-oriented dynamic neuromuscular stabilization system [23]. Therefore, the advantages of stability-oriented exercises remain unclear. Previous studies showed that DSS function might be improved by periodic strength training interventions in speed and strength for team disciplines, such as ice hockey and basketball [25,26]. However, their effectiveness in futsal is sufficiently recognized only in adolescent futsal players [27] and elite women [24], although recommendations for dynamic postural control development are available [28]. Since futsal games consist of complex movements and require high levels of agility, the development of the DSS in various body positions [29,30] should precede the use of heavyweight exercises [16,31,32]. The effect of stabilization-oriented exercises is expected but not experimentally verified.

Although current research provides some evidence about exercise selection to specific activation of the DSS [8,33,34], the DSS improvement after training intervention by stability-oriented exercises is rarely reported in the case of elite athletes. On the other hand, traditional exercises might have complex effects on muscle activation patterns if they are performed without movement pattern deviations. Therefore, this study aimed to compare the effect of traditional and stability-oriented strength exercises on trunk stability and DSS activation in elite futsal players.

## 2. Materials and Methods

### 2.1. Participants

Twenty elite futsal players from the Czech first league were randomly divided into an experimental intervention group (EG, n = 10, age 26 ± 8 years, height 182 ± 9 cm, weight 77 ± 17 kg) that performed a stability exercise program and a control group (CG, n = 10, age 27 ± 7 years, height 179 ± 14 cm, weight 78 ± 15 kg) that performed regular strengthening. No playing position and no relative age effect were considered during randomization since this issue remains unclear in elite futsal [22]. All participants had been on elite teams for at least 6 years, and their current habitual weekly training cycle met at least the following criteria: 6 training sessions per week, 120 min of technical-tactical training, 160 min of conditioning work (including intervention), 190 min of game time, and 130 min of warm-ups, similar as previous study [35]. All included participants were healthy and free of injury for three months before the participation in the study. The research and the informed consent form were approved by the institutional ethics committee of Charles University, Faculty of Physical Education and Sport (no 146/2019), following the ethical standards of the Helsinki Declaration of 2013. A signed written informed consent form was obtained from all participants in this study before the measurements were taken.

### 2.2. Design and Procedure

**Figure 1:**
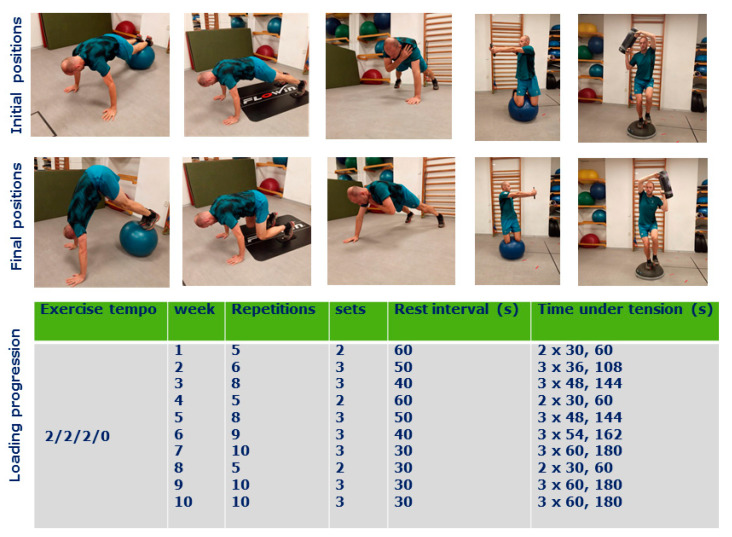
The stability-oriented exercises program (experimental group) performed at exercise tempo 2/2/2/0 with work-load progression. The main difference between the traditional exercises was in the position of the center of gravity or the stability of the surface.

**Figure 2:**
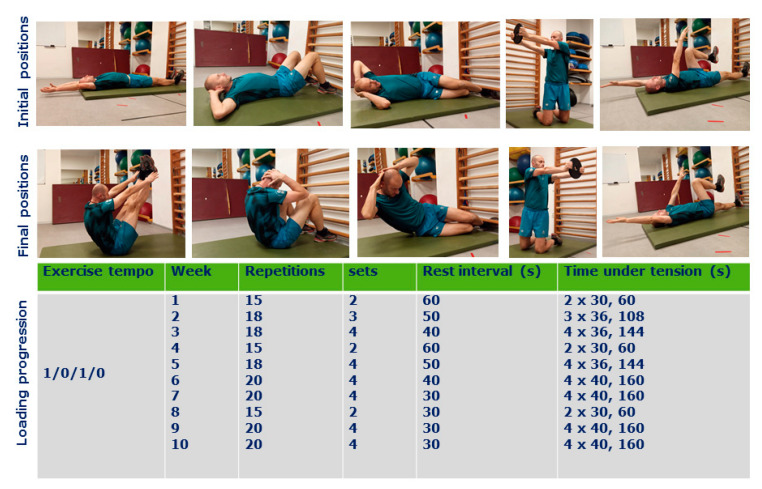
The traditional exercises program (control group) performed at exercises tempo 1/0/1/0 with work-load progression. The main difference between the stability-oriented exercises was in the position of the center of gravity or the stability of the surface.

Participants performed a total of 25 intervention sessions lasting for 30–40 min (including 10 min warm-up) over a period of 10 weeks as the first training of the day. Each intervention was performed two times a week during the 1st, 4th, 5th, 8th, and 10 weeks and three-time a week during the 2nd, 3rd, 6th, 7th, and 9th week. Both groups’ training consisted of exercises in varying body positions with a focus on keeping the trunk in a stabilized position in bilateral and unilateral fashions. The loading conditions were set up primarily for strength endurance using progressive loading increase (Figure 1 and Figure 2) according to Bompa [36]. If the external load was used, the weight was determined by the individual rate of perceived exertion scale 4–5, allowing to keep exercise technique during the last repetitions [22]. Both interventions were performed in the same gym facility by the players from the same team, where the conditioning coach (JS) was leading the training sessions. The participant was not allowed to use any food supplements or stimulants for the training sessions, nor to use any special diet. However, the exact diet content was not controlled during the study period, where athletes were maintaining their regular diet strategies.

The EG group performed stability-oriented exercises, including reverse sit-ups on a gym ball (Powerball Premium ABS–45 cm, Togu GmbH, Prien-Bachham, Germany) and slide board (FLOWIN PRO, Vintrie, Sweden), one arm planks, lateral arm raises on a gym ball with bodyweight or a light external load (Eleiko, Halmstad, Sweden), and one leg squats (to 80–90° knee flexion) on Bosu ball (Bosu ELITE, Ashland, OH, USA) with an aqua bag (Jordan, Kings Lynn, Norfolk, England) side switch (Figure 1), where each exercise was performed with voluntary, controlled contractions and a balanced tempo of movement phases of 2/1-2/2/0 (eccentric phase, isometric phase, concentric phase, rest between the repetitions). The isometric phase was set up for 2 s at initial repetitions with an allowed decrease to 1 s during later repetitions to maintain the exercise technique.

The CG performed traditional exercises consisting of sit-ups, side crunches, lateral arm, and rotations perpendicular to the floor contralateral, limb swings (Figure 2). If light external resistance was applied, a similar external load was applied in both groups, and exercises were performed with voluntary, controlled contractions, a balanced tempo of movement phases of 1/0/1/0, and swing allowed in isometric positions.

### 2.3. Measures of the Deep Stabilization System (DSS)

**Figure 3:**
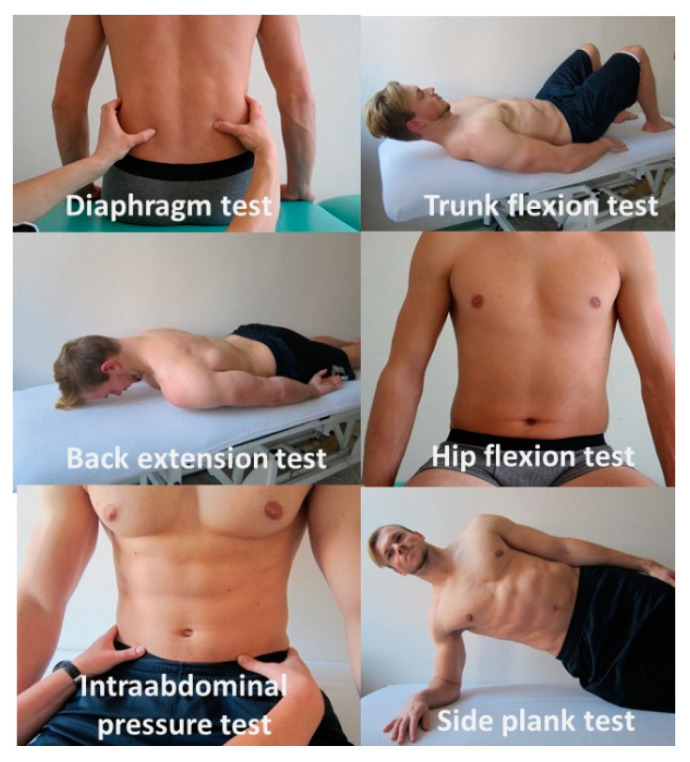
The battery of six deep stabilization tests.

There were three testing sessions during the study, before the intervention, in the 5th week of intervention (ongoing control measure), and after 10 weeks of DSS training. Each testing session was performed after 48 h rest period without any exhausting activity or DSS activation exercises. The DSS tests were performed first in the day before any warmup by inspection and palpation by one certified physiotherapist who was blinded to the participants’ type of intervention. This physiotherapist evaluated the function of the DSS on a five-point scale, where 1 was sufficient activity of the DSS, 2 was DSS activity with a lack of one activity function, 3 was DSS activity with a lack of several activity functions, 4 indicated an insufficient position hold, and 5 indicated insufficient DSS function. Each test was performed twice, while compliance result was used for the statistical analyses. The reliability of these methods is the same as that of other similar physiotherapy methods [37]. The six following DSS tests (Figure 3) were performed according to Kolar [11,12] in subsequent order: the diaphragm test, trunk flexion test, back extension test, hip flexion test, intraabdominal pressure test, and side plank test. Rest between the tests were minimum of the 30 s.

### 2.4. Diaphragm Test

The individual is assessed in a seated position in the upright posture, arms and legs relaxed, the chest is in a caudal or expiratory position. The examiner places fingers between and inferiorly to the patient’s caudal ribs and instructs the individual to take a deep breath and create counter-resistance toward the examiner’s fingers to activate the laterodorsal sections of the abdominal wall. The examiner assesses visually and by palpation any lateral movement of the lower ribs, the amount and symmetry of activation of the laterodorsal sections of the abdominal wall [12,38].

### 2.5. Trunk Flexion Test

The individual is assessed in the supine position, with arms relaxed along the trunk. Examiner instructs the individual to slowly flex the neck, followed by the trunk until the lower scapular angles come off the table. The examiner visually assesses the action of the thorax muscles [12,38].

### 2.6. Back Extension Test

The individual is assessed in the prone position, with arms relaxed along the trunk. Examiner instructs the individual to lift the head and do slide spine extension above the table. The examiner visually assesses the stabilization pattern from the side and from above (involved back and a lateral group of abdomen muscles) and may also palpate laterodorsal sections of the abdominal wall [12,38].

### 2.7. Hip Flexion Test

The individual is assessed in the seated position in an upright posture at the edge of the table, arms and legs relaxed, legs are without contact with the ground. Examiner instructs the individual to slowly alternately flex the hip (approximately 10–20 cm) above the table. The examiner visually assesses any spinal and pelvic movements and palpates laterodorsal sections of the abdominal wall and assesses the coordinated activity of the abdominal muscles [12,38].

### 2.8. Intraabdominal Pressure Test (IAP Test)

The individual is assessed in the seated position in an upright posture, arms and legs relaxed. Examiner palpates the lower abdominal sections above the groin (medially from the anterior superior iliac spine and the femoral heads of hip joints) and instructs the individual to activate the abdominal wall and create intra-abdominal pressure by pushing against the examiner’s fingers placed above the inguinal ligaments. The examiner assesses the amount and symmetry of activation while visually observing the abdominal contour and any umbilicus movement at the same time [12,38].

### 2.9. Side Plank Test

The individual is assessed in a side plank position; the lower arm is supported on the forearm, the upper arm is placed relaxed on the homolateral hip. The examiner visually assesses the action of the abdominal and thorax muscles and also assesses the coordinated action of shoulder gilder muscles at the same time [18].

### 2.10. Statistical Analysis

Due to the ordinal nature of the data and sample size, we used a non-parametric statistic, and the median and 25th and 75th percentiles were used to describe repeated measurements of DSS activation. Data normality was tested by the Kolmogorov-Smirnov test. Friedman ANOVA was used to assess changes in repeated measurements of individual groups. Differences between groups at the beginning and end of the intervention were assessed by Kruskal-Wallis ANOVA. If significant intergroup differences were found, pair differences were verified by a nonparametric Mann-Whitney test. The significance level was set to *p* ≤ 0.05, where a combination of significant pre and post difference between group difference was considered as the main outcome. All calculations were processed by IBM SPSS for Windows statistical software (version 24, Chicago, IL, USA).

## 3. Results

**Figure 4:**
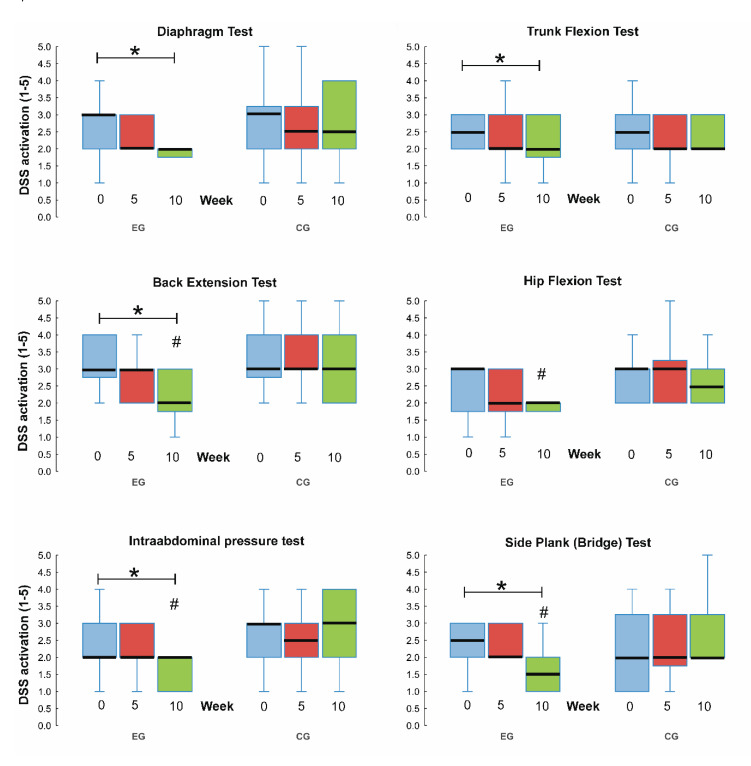
Results of the deep stabilization system tests before intervention (0), after 5 weeks of intervention (5), and after completing 10 weeks of intervention (10) in the control (CG) and experimental group (EG). * Significant differences between repeated measures within each group by Friedman ANOVA (*p* < 0.05). # significant difference between the CG and EG by Kruskal-Wallis ANOVA (*p* ≤ 0.05). DSS = dynamic stabilization system. The boxplot shows median (black line), lower and upper quartile (column), and minimal and maximal values (whiskers).

No intergroup differences were found in DSS activation at the beginning of the intervention, indicating the same starting condition for all groups (Figure 4), and data normality was disrupted according to the Kolmogorov–Smirnov test. Friedman ANOVA showed that the EG improved significantly in all tests except for hip flexion (Figure 4). There were no significant changes in DSS activation in the CG. Kruskal-Wallis ANOVA showed intergroup differences for the intraabdominal pressure test (*p* = 0.004), side plank (0.002), and back extension test (*p* = 0.036) between groups after 10 weeks of intervention (Figure 4). The test for hip flexion was at the limit of significance (*p* = 0.055).

## 4. Discussion

The main finding of this study showed that stability-oriented exercises have a direct effect on the DSS, while traditional exercises do not, which is in accordance with the current recommendations for athlete conditioning [10,16,39,40,41]. The success of the presented method relies not only on using unstable positions but also on motor control of the speed of the contractions and the contraction range of motion. Thus, there are several motor control differences between DSS and traditional exercises, which causes detectable effects on DSS function. On the other hand, the absence of DSS exercises might cause maintenance or the development of muscle imbalances, which can cause DSS dysfunction. Along with the DSS function, the suggested program supports the player’s performance in functional movements, as it was proved in previous studies [23,24].

The presented program was in accordance with recommendations for developing dynamic balance in futsal players, including exercises for hip and knee flexion and dynamic core stability in the frontal plane [28] and other directions. Although futsal is known for high injury rate (196 injuries per 1000 player match hours) [1], we haven’t recorded serious injury-causing training interruption during our intervention period. Therefore, we could state that this program has the potential to increase players’ conditioning for field performance as well as injury prevention. Moreover, since this program is based on a selection of exercises focused on core training [8,33,42,43], it should be incorporated into training programs, such as those for strengthening the core muscles and the whole DSS. Since another injury prevention program, the “FIFA 11+” warm-up, has been reported to be questionably effective [27,44] and possibly ineffective [34] for the development of futsal player proprioception, we suggest studying the interaction of the DSS with FIFA 11+ to determine whether their effects interfere.

Based on the lack of improvement with regular strengthening exercises, we conclude that complex movements during futsal do not develop trunk stability by activating the DSS; thus, the futsal players have to include DSS training to avoid the development of muscle imbalances. Other training interventions, such as training on an unstable surface, do not necessarily lead to improvement of the DSS [45,46,47], especially in elite athletes [45]. DSS exercise seems to be a good alternative to the unstable surface, which has been shown to improve functional movement screen in elite female futsal players [24]. It has been documented that the absence of preventive strength exercises decreases the effectiveness of whole conditioning programs [12,48] and might cause muscle imbalances [16]. A previous study [25] performed on elite ice hockey players concluded that a training intervention focusing on DSS muscles might eliminate pain in the pelvic region, condition the DSS muscles, and influence posture. On the other hand, traditional exercises, such as sit-ups and pushups, do not stimulate the DSS in elite athletes and do not prevent muscle imbalances unless they are performed in combination with DSS stimulation.

The study limitations are in the comparison of only two groups without a crossover design, possibly causing us to observe the same effect in all participants. On the one hand, there is a presumption that players with already functioning DSSs would strengthen their DSS during complex movements and thus might not have decreased in DSS function even after a training period without continuing DSS-specific training. However, this hypothesis should be studied in further research, as well as the possible effect of playing position. Another limitation also relies on the fact that the players did not have regular resistance training and other corrections of joint centration during the study period, which might have increased the effectiveness of both kinds of training. However, this highlights the efficiency of DSS exercises and there being an effective tool. One of the limitations is the reliability of the physiotherapy assessment, as it has only been performed by a single practitioner.

## 5. Conclusions

Stability-oriented exercises had a greater effect on DSS function than traditional strength exercises. Stabilization-oriented exercises effectively activate the function of the deep stabilization system; therefore, those exercises should have priority over traditional strength exercises in injury prevention training programs. The use of stabilization-oriented exercises might prevent injury and overloading in elite futsal players or in players of other sport games, which are typical by asymmetrical loading caused by lateral preference. The DSS is a sufficient injury-preventive program to use 2–3 times a week in competition season of elite adult futsal players.

## References

[1] Junge A., Dvorak J. (2010). Injury risk of playing football in Futsal World Cups. Br. J. Sports Med..

[2] Varkiani M.E., Alizadeh M.H., Pourkazemi L. (2013). The epidemiology of futsal injuries via sport medicine federation injury surveillance system of Iran in 2010. Procedia-Soc. Behav. Sci..

[3] Naser N., Ali A., Macadam P. (2017). Physical and physiological demands of futsal. J. Exerc. Sci. Fit..

[4] De Freitas V.H., Ramos S.d.P., Leicht A., Alves T., Rabelo F., Bara-Filho M.G., Guarnier F.A., Nakamura F.Y. (2017). Validation of the futsal-specific intermittent shuttle protocol for the simulation of the physical demands of futsal match-play. Int. J. Perform. Anal. Sport.

[5] Ayarra R., Nakamura F.Y., Iturricastillo A., Castillo D., Yanci J. (2018). Differences in physical performance according to the competitive level in futsal players. J. Hum. Kinet..

[6] Ribeiro J.N., Gonçalves B., Coutinho D., Brito J., Sampaio J., Travassos B. (2020). Activity Profile and Physical Performance of match play in elite futsal players. Front. Psychol..

[7] Small K., McNaughton L., Greig M., Lovell R. (2010). The effects of multidirectional soccer-specific fatigue on markers of hamstring injury risk. J. Sci. Med. Sport.

[8] Martuscello J.M., Nuzzo J.L., Ashley C.D., Campbell B.I., Orriola J.J., Mayer J.M. (2013). Systematic review of core muscle activity during physical fitness exercises. J. Strength Cond. Res..

[9] Bryan M., Hawson S. (2003). The benefits of Pilates exercise in orthopaedic rehabilitation. Tech. Orthop..

[10] Jebavý R., Hojka V., Kaplan A. (2017). Kondiční Trénink ve Sportovních Hrách: Na Příkladu Fotbalu, Ledního Hokeje a Basketbalu.

[11] Frank C., Kobesova A., Kolar P. (2013). Dynamic neuromuscular stabilization & sports rehabilitation. Int. J. Sports Phys..

[12] Kolář P. (2009). Rehabilitace v Klinické Praxi.

[13] Suchomel R.f.M.T. (2006). Stabilita v pohybovém systému a hluboký stabilizační systém. Rehabil. Fy..

[14] Daggfeldt K., Thorstensson A. (1997). The role of intra-abdominal pressure in spinal unloading. J. Biomech..

[15] Blazek D., Stastny P., Maszczyk A., Krawczyk M., Matykiewicz P., Petr M. (2019). Systematic review of intra-abdominal and intrathoracic pressures initiated by the Valsalva manoeuvre during high-intensity resistance exercises. Biol. Sport.

[16] Hodges P.W. (2003). Core stability exercise in chronic low back pain. Orthop. Clin..

[17] Pysna J., Pysny L., Petru D., Endal V. (2018). Evaluating activity of deep stabilising system of the spine in young elite ice-hockey players. South. Afr. J. Res. SportPhys. Educ. Recreat..

[18] Jalovcova M., Pavlů D. (2010). Stabilizační systém a role m. transversus abdominis. Rehabil. Fyzikální Lékařství.

[19] Long A., Donelson R., Fung T. (2004). Does it matter which exercise? A randomized control trial of exercise for low back pain. Spine.

[20] Aspe R.R., Swinton P.A. (2014). Electromyographic and kinetic comparison of the back squat and overhead squat. J. Strength Cond. Res..

[21] Cordo P.J., Gurfinkel V.S., Smith T.C., Hodges P.W., Verschueren S., Brumagne S. (2003). The sit-up: Complex kinematics and muscle activity in voluntary axial movement. J. Electromyogr. Kinesiol..

[22] Jahoda R., Mitterbauer G. (2013). ComplexCoreTM-Core Stabilisation in Training and Therapy.

[23] Mahdieh L., Zolaktaf V., Karimi M.T. (2020). Effects of dynamic neuromuscular stabilization (DNS) training on functional movements. Hum. Mov. Sci..

[24] Lago-Fuentes C., Rey E., Padrón-Cabo A., de Rellán-Guerra A.S., Fragueiro-Rodríguez A., García-Núñez J. (2018). Effects of core strength training using stable and unstable surfaces on physical fitness and functional performance in professional female futsal players. J. Hum. Kinet..

[25] Pešán F., Jelínek M., Fiala M., Matošková P., Süss V. (2015). Vliv kompenzačního programu na posturální svaly u extraligových hráčů ledního hokeje. Rehabilitácia.

[26] Ondra L., Nátěsta P., Bizovská L., Kuboňová E., Svoboda Z. (2017). Effect of in-season neuromuscular and proprioceptive training on postural stability in male youth basketball players. Acta Gymnica.

[27] Busara J., Chentanez T., Pintong M., Widjaja W. (2015). The effects of the 11+ training programme on stability performance in adolescent futsal players. J. Sports Sci. Technol..

[28] Ruiz-Pérez I., Ayala F., Puerta J.M., Elvira J.L.L., De Ste Croix M., Hernández-Sánchez S., Vera-Garcia F.J. (2019). A Bayesian Network approach to study the relationships between several neuromuscular performance measures and dynamic postural control in futsal players. PloS ONE.

[29] Bačová I., Cicholesová T., Dziaková M., Šulla I., Kitka M., Petrovičová J. (2015). Význam Rehabilitácie Hlbokého Stabilizačného Systému Pri Liečbe Vertebrogénnych Ochorení.

[30] Šorfová M., Tlapáková E., Matějková A. (2018). Funkce svalů pánevního dna ve vztahu k poloze těla a k typu dýchání. Rehabil. Phys. Med./Rehabil. Fyzikalni Lek..

[31] Siff M. (2003). Supertraining.

[32] Zatsiorsky V., Kraemer W. (2006). Science and Practice of Strength Training.

[33] Willardson J.M. (2007). Core stability training: Applications to sports conditioning programs. J. Strength Cond. Res..

[34] Lopes M., Lopes S., Patinha T., Araújo F., Rodrigues M., Costa R., Oliveira J., Ribeiro F. (2019). Balance and proprioception responses to FIFA 11+ in amateur futsal players: Short and long-term effects. J. Sports Sci..

[35] Stastny P., Lehnert M., De Ste Croix M., Petr M., Svoboda Z., Maixnerova E., Varekova R., Botek M., Petrek M., Kocourkova L. (2019). Effect of COL5A1, GDF5, and PPARA Genes on a Movement Screen and Neuromuscular Performance in Adolescent Team Sport Athletes. J. Strength Cond. Res..

[36] Bompa T., Buzzichelli C. (2015). Periodization Training for Sports, 3E.

[37] Cejudo A., de Baranda P.S., Ayala F., Santonja F. (2015). Test-retest reliability of seven common clinical tests for assessing lower extremity muscle flexibility in futsal and handball players. Phys. Sport.

[38] Kobesova A., Davidek P., Morris C.E., Andel R., Maxwell M., Oplatkova L., Safarova M., Kumagai K., Kolar P. (2020). Functional postural-stabilization tests according to Dynamic Neuromuscular Stabilization approach: Proposal of novel examination protocol. J. Bodyw. Mov..

[39] Richardson C.A., Hodges P., Hides J.A. (2004). Therapeutic exercise for lumbopelvic stabilization: A motor control approach for the treatment and prevention of low back pain. Phys. Ther..

[40] Arokoski J.P., Valta T., Airaksinen O., Kankaanpää M. (2001). Back and abdominal muscle function during stabilization exercises. Arch. Phys. Med. Rehabil..

[41] Dallas G., Mavvidis A., Kirialanis P., Papouliakos S. (2017). The effect of 8 weeks of whole body vibration training on static balance and explosive strength of lower limbs in physical education students. Acta Gymnica.

[42] Stephenson J., Swank A.M. (2004). Core training: Designing a program for anyone. Strength Cond. J..

[43] Willardson J.M. (2007). Core stability training for healthy athletes: A different paradigm for fitness professionals. Strength Cond. J..

[44] Gritsanadilok W., Chentanez T., Hirunrat S., Sinphurmuksakul O. (2013). The effect of “The FIFA 11+” Warm-up training on balance and proprioception in adolescent futsall players. J. Sports Sci. Technol..

[45] Wahl M.J., Behm D.G. (2008). Not all instability training devices enhance muscle activation in highly resistance-trained individuals. J. Strength Cond. Res..

[46] Jebavy R., Balas J., Jalovcova M. (2016). Komparace silových cvičení na nestabilních a stabilních plochách jako prostředek pro zlepšení činností hlubokého stabilizačního systému. Rehabilitácia.

[47] Jebavý R., Perič T., Baláš J., Šťastný P. (2013). Stimulation a strength endurance through exercises on the unstable surfaces. Studia KinanthropologicaUniv. Bohem. Merid. Budvicensis.

[48] Yaggie J.A., Campbell B.M. (2006). Effects of balance training on selected skills. J. Strength Cond. Res..

